# Redox Regulation of Cysteine-Dependent Enzymes in Neurodegeneration

**DOI:** 10.1155/2012/703164

**Published:** 2012-07-05

**Authors:** Rodney P. Guttmann, Tamara J. Powell

**Affiliations:** Center on Aging, School of Psychological and Behavioral Sciences, University of West Florida, 11000 University Parkway, Pensacola, FL 32514, USA

## Abstract

Evidence of increased oxidative stress has been found in various neurodegenerative diseases and conditions. While it is unclear whether oxidative stress is a cause or effect, protein, lipid, and DNA have all been found to be susceptible to oxidant-induced modifications that alter their function. Results of clinical trials based on the oxidative-stress theory have been mixed, though data continues to indicate that prevention of high levels of oxidative stress is beneficial for health and increases longevity. Due to the highly reactive nature of the sulfhydryl group, the focus of this paper is on the impact of oxidative stress on cysteine-dependent enzymes and how oxidative stress may contribute to neurological dysfunction through this selected group of proteins.

## 1. Introduction

It is clear that while oxygen is essential for life in order to produce chemical energy in the form of ATP, paradoxically, the byproduct of its metabolism generates multiple reactive oxygen species (ROS) that are associated with cellular toxicity. Specifically, in regards to neurodegeneration, there is substantial evidence that ROS are a major component of diseases including Alzheimer's, Parkinson's, and amyotrophic lateral sclerosis [1–4]. While clinical trials aimed at decreasing the burden of oxidative stress have not clearly demonstrated effectiveness, genetic research has found that high levels of antioxidant enzymes prolong life and decrease pathology. In addition, animal models have also indicated that oxidative stress is an important and consistent characteristic of many forms of neurodegeneration.

One particular group of proteins that appear to be intimately involved in the neurodegenerative processes is the cysteine-dependent proteins. This group includes various proteases, antioxidant enzymes, kinases, phosphatases, and other types of enzymes as well as other nonenzymatic proteins such as those that use cysteine as a structural component rather than as part of a catalytic site. More research will be needed to firmly establish the extent to which oxidative stress is causal in these diseases, but based on current understanding, therapies to reverse the oxidant-induced modifications of proteins, lipids or, DNA are expected to be beneficial. This paper will highlight some selected, yet significant cysteine-dependent enzymatic systems that rely on a proper redox environment for their activity and provide evidence for their redox control in neurodegenerative disease. Potential relationships to cancers will also be discussed.

## 2. Redox Sensitivity of Cysteine

**Figure 1:**
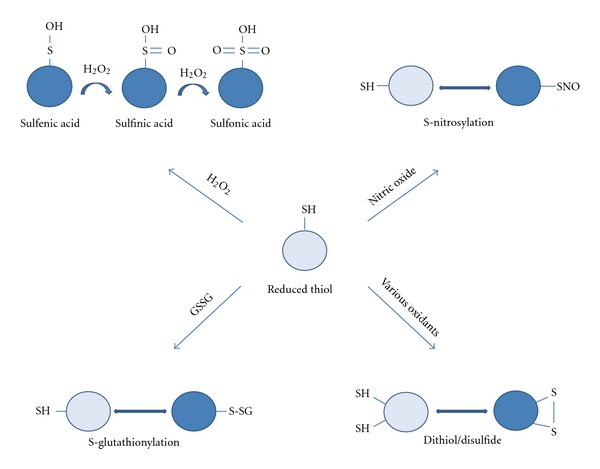
Diagrammatic representation of major oxidation states of cysteine that have been found in vivo. Circles represent a protein that contains a cysteine within its primary structure. In its most reduced state, the sulfur group of cysteine is found in the form of –SH. The sulfur can become modified in a number of ways including S-nitrosylated by nitric oxide or S-glutathionylated by glutathione, which are being increasingly recognized for their importance in regulated many cysteine-containing enzymes. In addition, the sulfur group can be oxidized to sulfenic, sulfinic, and sulfonic acids or it may form an intra- or inter-molecular disulfide bond.

The aminoacid cysteine is highly sensitive to redox state. This is largely due to the reactivity of anionic sulfur to various oxidizing agents that can form multiple types of oxidized species (see Figure 1). However, not all cysteines are equally sensitive, and such sensitivity has been utilized throughout evolution to provide protection against oxidative stress.

A close examination of the variety of physiologically occurring antioxidant systems, that use cysteine as a major component of their antioxidant activity or as part of a redox “sensor,” clearly demonstrates the sensitivity and evolutionary significance of cysteine as part of a protein's active center [5]. For example, glutathione (GSH) consists of glutamate, glycine, and cysteine and is the major antioxidant found in brain. It is found at millimolar levels and is a major determinant of intracellular redox conditions. Cysteine itself has been shown to be the major extracellular antioxidant. Further examples of cysteines' critical role in redox balance can be found in other enzymatic systems including the multiple enzymes involved in the maintenance of peroxiredoxins, glutaredoxins, and thioredoxins among others. The natural role of cysteines as redox sensors is further observed by the observation that throughout evolution, cysteines are found in transcriptional regulators that are modulated by oxidative stress such as oxyR and Nrf2/Keap [6].

Due to the varying microenvironments that exist for cysteine within a given protein structure, cysteines are not equally reactive. For example, as discussed further below, the Parkinson's disease-linked protein, DJ-1 cysteine at position 106, appears to be highly sensitive to oxidative attack, while two other cysteines within its structure are not as easily modified [7]. Such apparent specificity of cysteines within the same protein is also observed among many other proteins [8, 9]. In terms of the macroenvironment, cysteine-dependent enzymes require a reducing environment for activity, which is the condition maintained in the cytoplasm in contrast to the extracellular space that is oxidizing. However, the lysosomal compartment is variable, and changes in redox state have been shown to modulate enzymatic activities located within it. For example, cathepsin activity was found to be altered through redox state as detected by a change in cleavage pattern produced under varying redox conditions [10]. Thus, the location of the cysteine within the overall tertiary/quaternary structure as well as its macroenvironment (e.g., intracellular versus extracellular or organelle) plays major roles in the extent to which a cysteine is stabilized in the anionic transition state, thereby affecting its reactivity to a change in the redox state.

## 3. Sources of Oxidants in Brain

Environmental toxins are thought to be a significant contributor to neuronal-related disorders including AD and particularly PD. These include a variety of naturally occurring and synthetic compounds, which results in the production of reactive species through well-characterized chemical pathways including the Fenton reaction and others [11]. In addition to direct chemical means, many of these environmental molecules, such as rotenone or paraquat, target mitochondria and disrupt the efficient production of energy, leading to abnormal increases in free radical production such as superoxide [12]. The identification of these toxins and their mechanisms of action is the subject of extensive research with a major emphasis on how their toxicity relates to the production of free radicals.

Besides environmental toxins, there are also important cellular sources of oxidants localized within cytosolic and mitochondrial compartments. Cellular sources include NADPH oxidases, which are enzymes associated with both signal transduction and the killing of foreign organisms through the production of superoxide. Monoamine oxidase (MAO) located at the mitochondrial surface is also a source of hydrogen peroxide. Due to MAO-B's role in the metabolism of dopamine, MAO activity is linked to PD in part due to the production of reactive oxygen species resulting from MAO-mediated metabolism of dopamine [13].

Endogenous superoxide production is strongly associated with the mitochondria and can occur within the matrix, the intermembrane space, and at the outer membrane of mitochondria. For example, reactive species are formed as part of electron transport including complex I (NADH-ubiquinone oxidoreductase). Complex I is considered to be an important source of free radical generation [14, 15] and does so during either forward electron flow or reverse electron transport [16]. Though debate exists about the mechanisms involved (one-site versus two-site model), the importance of superoxide and hydrogen peroxide formation through the various mitochondrial pathways should not be underestimated as oxidants produced through the mitochondria are considered highly relevant to aging and neurodegeneration.

## 4. Oxidative Stress in Neurodegenerative ****Disease

Over the last few decades it has become increasingly clear that the human brain is more sensitive to various forms of oxidative attack damage compared to other organs in the body. This is due in large part to the high metabolic activity found in brain and the seemingly limited capacity for the repair of damage to neurons as a result of injury. Many types of oxidizing molecules have been observed in the human brain, and their presence is associated with selective damage to brain regions linked with neurodegenerative disease. While it is uncertain as to the extent in which the increase in reactive species causes the visible pathological hallmarks, the formation of reactive oxygen, nitrogen, or sulfur species is nevertheless generally recapitulated in animal models of each disease, strongly suggesting a potential causal link. Observed biomarkers of increased oxidative stress include 4-hydroxynonenal, thiobarbituric acid-reactive substances, free fatty acid release, and acrolein formation for lipid peroxidation; 8-hydroxy-2-deoxyguanosine for DNA; protein carbonyls, 3-nitrotyrosine, and glutathionylation for proteins. In regards to proteins, along with cysteine, multiple aminoacids are found to be modified including lysine, methionine, histidine, and others.

### 4.1. Alzheimer's Disease

AD is an age-associated progressive neurodegenerative disease that affects behavior, cognition, and memory and is characterized by two major pathological hallmarks: extracellular plaques composed primarily of A*β* and intracellular inclusions of tau protein known as tangles. It currently has no known cause or cure and remains the most common form of irreversible dementia affecting approximately 20 million people worldwide. Oxidative damage is one of the earliest detectable changes observed in both genetic and sporadic forms of Alzheimer's disease [17].

While there are several theories about the source of the various oxidizing molecules, A*β* has been a prime candidate. Indeed, treatment of various model systems with different A*β* forms typically results in increased oxidative stress. Recent work has shown that extracellular A*β* treatment results in atypical redox effects in astrocytes compared to treatment with other oxidizing molecules, suggesting that A*β* possesses unique oxidizing properties [18]. In addition to the potential for A*β* to stimulate increased oxidative stress, there is also evidence that major antioxidant systems such as superoxide dismutase, catalase, and others have decreased activity associated with AD progression [19].

#### 4.1.1. Examples of Oxidized Enzymes in AD

 Peroxiredoxins (Prxs) are a family of peroxidases that reduce peroxynitrite and a variety of other hydroperoxides. They use a redox-sensitive cysteines within their active site reducing the peroxide substrates either through the formation of an intramolecular disulfide bond or oxidation to sulfinic acid or sulfonic acid [20]. Proteomic studies for subjects with early AD found that Prx-2 was oxidized in a brain region containing significant AD-related pathology compared to age-matched controls [21]. In another study by Cumming and colleagues [22], it was not only shown that Prx-2 was more oxidized in AD brains, but also treatment of cultured primary neurons with A*β* resulted in Prx oxidation that was reversible by addition of a cysteine-specific antioxidant, N-acetylcysteine. In addition, Fang and colleagues found that Prx-2 was S-nitrosylated at active-site cysteines Cys 51 and Cys 172 [23].

Protein disulfide isomerase (PDI) is a multifunctional enzyme with several family members. These enzymes include chaperone activity mediated by catalyzing the reduction, oxidation, and isomerization of protein disulfides to maintain proper protein folding. PDI redox activity is based on the presence of two thioredoxin-like motifs (CXXC) (human PDI: Cys 36/39 and Cys380/383). It has been found to be oxidized in AD and colocalizes with neurofibrillary tangles [24]. Though no changes in the amounts of PDI have been noted in AD brain, Uehara and colleagues [25] did show that PDI was S-nitrosylated at multiple cysteines in AD brain and that such oxidation resulted in enzyme inactivation. Since PDI is important for the folding of proteins by catalyzing cysteine-disulfide exchange, its inactivation increased the levels of misfolded of proteins, leading to the activation of the unfolded protein response.

Calpains are calcium and cysteine-dependent endoproteases whose active-sites are sensitive to oxidative inactivation. In addition to AD, calpains play a role in multiple disease states, including cancer [26, 27]. As a putative physiological regulator of key proteins associated with AD such as amyloid precursor protein and tau among others, understanding calpains' potential dysregulation by redox status is important. Calpain's active site cysteine (Cys105) was found to be oxidized, in vitro and in cultured cells only in the presence of calcium [28, 29]. Presumably, this is because the active site is otherwise inaccessible to oxidative attack when the enzyme is in an inactive conformation. Evidence suggests that calpain-like enzymatic activity is also inhibited in brain regions of AD associated with high pathology [30].

### 4.2. Parkinson's Disease

Parkinson's disease is the second most common neurodegenerative disease characterized by loss of dopaminergic neurons, glutathione depletion, oxidative stress, and the formation of intracellular inclusions of alpha-synuclein called Lewy bodies. Similar to AD, the vast majority of PD cases are sporadic with only 5–10% of cases due to genetic causes [31]. Much of what we understand has been gained through the use of animal models of PD that involve the administration of exogenous compounds such as 1-methyl-4-phenyl-1,2,3,6-tetrahydropyridine (MPTP), rotenone, and paraquat. Investigation of these compounds has strongly linked them to mitochondrial dysfunction and the abnormal production of free radicals, which generally reflects what is observed in the human disease. In the course of these studies, several highly relevant cysteine-dependent enzymes known to contribute to PD have been observed to be modified at key cysteine residues by these reactive species.

As discussed above, PDI has also been found to be oxidized in samples of PD brain [25]. The potential impact of PDI in PD brain is evidenced by experiments suggesting that PDI plays a role in the folding of both synphilin-1 and alpha-synuclein [25, 32], two proteins closely linked to PD. Other studies have also found links between PDI oxidation and PD [33, 34].

DJ-1 activity is also altered by oxidative stress. DJ-1 is a 20 kDa that has multiple putative activities as a protease and an antioxidant among others [35]. DJ-1 is strongly associated with PD because mutations in DJ-1 result in autosomal recessive early-onset form of Parkinson's disease. DJ-1 contains three cysteine residues that each has been evaluated in response to oxidative stress. Based upon multiple studies, it is clear that only Cys-106 oxidation is an important regulatory component for DJ-1 activity. For example, Waak and colleagues [36] found that the formation of a mixed disulfide, created under oxidizing conditions between DJ-1 and apoptosis signal-regulating kinase 1 (ASK1), contributes to DJ-1′s neuroprotective effects. Such protective effects are predicted to be lost in cases that may occur with aging or exposure to oxidative toxins such as those used in animal models of PD including MPTP or rotenone.

Another example of a cysteine-containing enzyme that is modified in PD is parkin. Parkin is an ubiquitin E3 ligase that serves to ubiquitinate a series of proteins and contains multiple cysteines that are required for full activity. Mutation of parkin is responsible for early-onset autosomal recessive juvenile Parkinsonism. Several studies have convincingly demonstrated that parkin is S-nitrosylated in cases of PD as well as in model systems [37, 38]. Such oxidation inhibits parkin's ubiquitin E3 ligase activity and therefore prevents proper ubiquitination of its substrates leading to accumulation of misfolded proteins. In addition, it also appears to be sensitive to covalent modification by dopamine itself [39].

Tyrosine hydroxylase (TH) is the initial and rate-limiting step in the biosynthesis of the dopamine (DA) and norepinephrine. This enzyme contains seven cysteines some of which have been found to be important for full TH activity. Kuhn and colleagues [40] found that 4-5 cysteines were modified by quinone derivatives of DOPA, dopamine, and N-acetyldopamine that were prevented by various thiol-reducing agents. Further, they found that such oxidations resulted in inhibition of TH enzymatic activity. Other evidence of TH redox sensitivity comes from Sadidi et al., [41] who found that peroxnitrite and nitrogen dioxide both inhibited TH through nitration of cysteines or through S-thiolation in the presence of GSH or cysteine. Additional discussion of redox regulation of TH can be found in a recent, excellent review [42].

### 4.3. Amyotrophic Lateral Sclerosis

More commonly known as Lou Gehrig's disease, ALS is the most common degenerative disease of the motor neuron system that results in the death of motor neurons, causing muscle weakness and eventually death. Despite an annual incidence rate of one-to-two cases per 100,000, the etiology of the disease remains largely unknown [43]. Although multiple theories have been presented, research focusing on neurotoxicity has revealed excessive entry of glutamate into the neurons damages cell metabolism, resulting in pathologic changes [43]. It has been offered that ALS develops when vulnerable persons are exposed to a neurotoxin at times of strenuous physical activity [44].

#### 4.3.1. Examples of Oxidized Enzymes in ALS

Mutations in Cu/Zn superoxide dismutase gene (SOD1) are associated with familial amyotrophic lateral sclerosis. Recent work has found that oxidative modification of SOD1 results in the formation of an epitope consistent with misfolding of SOD1 that is observed in ALS [45]. Subsequently, Redler and colleagues [46] evaluated the effects of specific modification of Cys-111 on this conformation change and found oxidation of Cys-111 via glutathionylation (see Figure 1) resulted in the destabilization of the SOD1 dimer. This destabilization increases the potential for unfolding of the monomer and subsequent aggregation, leading to loss of SOD1 activity and promoting cell death.

## 5. Other Cysteine-Dependent Enzymes Affected by Oxidative Stress

Beyond cases clearly associated with specific disease pathology, there are other physiologically regulated or pathologically modified cysteine-dependent enzymes that are equally important to consider and have been suggested to play a role in neurodegeneration. For example, Janus kinase 2 (JAK2) is part of the JAK2/signal transducers and activators of Transcription pathway that plays a role in synaptic plasticity, cell proliferation, migration, and apoptosis. JAK2 contains a pair of cysteine residues (Cys866 and Cys917) that act as a redox-sensitive switch for its activity [47] and was shown to be inactivated by treatment of human BE (2)-C neuroblastoma cells with rotenone, a chemical used to model PD in animals [48].

Members of the caspase family are also found to be regulated by redox state. Caspases are involved in the initiation and execution of certain forms of programmed cell death and are therefore linked to multiple neurodegenerative conditions. Several studies have confirmed that members of this group can be oxidized at their active-site cysteine through S-nitrosylation, resulting in enzyme inhibition [49–51]. However, there are other examples in which nitric oxide (NO) may activate these caspases [52]. Such discrepancies are likely due to duration and dose of NO as well as other indirect effects of NO on other activation mechanisms [53].

 Phosphatase and tensin homolog (PTEN) dephosphorylates phosphatidylinositol (3,4,5)-trisphosphate (PIP_3_) to phosphatidylinositol (4,5)-bisphosphate (PIP_2_), serving to antagonize the kinase activity of phosphatidylinositide-3-kinase. As part of this pathway that includes the Akt cascade, PTEN activity is relevant to apoptosis. Numajiri and colleagues [54] reported that S-nitrosylation of PTEN at Cys-83 inhibited PTEN activity resulting in increased AKT activity downstream, promoting cell survival. Interestingly, they also found that at higher levels of NO, AKT itself could be S-nitrosylated and therefore inhibited, resulting in a pro-apoptotic environment [54]. Finally, the active-site cysteine of PTEN, Cys124, has also been found to be oxidized in the presence of high concentrations of hydrogen peroxide [55].

Glyceraldehyde 3-phosphate dehydrogenase (GAPDH) is a glycolytic enzyme that also has other recently discovered roles including participating in apoptosis. Cumming and Schubert [56] showed that GAPDH is sensitive to oxidative stress in affected brain regions of AD. They reported an increase in GAPDH intermolecular disulfide formation that was reversed by addition of the cysteine-specific reducing agent, dithiothreitol (DTT), that included the active-site Cys149. Treatment of cultured neuronal and neuronal-like cells with A*β* also resulted in GAPDH oxidation in addition to nuclear translocation and aggregation that may contribute to apoptosis [56]. As GAPDH's role is more fully elucidated, its oxidation is likely to be discovered to reach beyond what is presently known. See Table 1 for examples of cysteine-dependent enzymes that have been found to be regulated by redox state within the various categories of enzymes.

## 6. Cysteine-Dependent Enzymes and Their Link to Cancer

Given the linkage between age and cancer, there are likely important connections between redox regulation of the enzymes associated with neurodegeneration discussed above and tumor formation. Indeed, there are many examples of cysteine-dependent enzymes playing important roles in the various aspects of cancer progression including the impact of cancer therapies on these enzymes. The following is a brief summary of examples of this overlap.

 Wang and colleagues [57] found in their model using MCF-7 human breast cancer cells that became resistant to radiation, that Prx2 is upregulated and may be a contributing factor to resistance to radiation. They hypothesized that this may be due to the antioxidant function of Prx2, resulting in attenuating radiation-induced oxidative stress effects. Goplen and colleagues [58] found that PDI is highly expressed during glioma invasion and that treatment with bacitracin, or a monoclonal antibody to PDI, inhibited tumor migration and invasion. Calpain-2 has been shown to play a role in calcium-dependent glioblastoma invasion, but not migration, which may be related to calpain-2′s function in invadopodial dynamics mediated by its regulation of matrix metalloproteinase 2 [59, 60]. DJ-1 appears to be upregulated in multiple forms of cancer and is considered a ras-dependent oncogene [61, 62]. DJ-1 upregulation, as with Prx2, is likely due to its antioxidant properties and the protective effects it would convey upon tumor cells. Alterations of parkin, observed in multiple cancer types, with genetic or other causes of decreased parkin levels are linked to increased tumorigenesis. As recently reviewed, and epidemiological studies suggest, parkin, along with DJ-1 and other genetically linked proteins are under investigation with respect to increasing risk of melanoma in PD [63–67]. SOD1, by virtue of its potent antioxidant activity, has been identified as a potential drug target to induce cell death in certain cancers. Somwar and colleagues [68], using a lung adenocarcinoma cell line, found that inhibition of SOD-1 led to increased apoptosis in these cells. Finally, Joshi and colleagues [69] treated mice with adriamycin, a chemotherapeutic agent, resulting in increased oxidation of Prx1, a cysteine-dependent peroxiredoxin, in brain.

From these data, two observations can be made. First, there are multiple cysteine-dependent enzymes that are sensitive to oxidative stress linked to tumor formation or migration. Second, the oxidizing effects of chemotherapeutic agents such as adriamycin should be considered when evaluating the potential effects of these compounds in terms of both their therapeutic and pathological effects.

## 7. Summary

Clearly, the redox regulation of cysteine-dependent enzymes is an important area of study. This is particularly evident in neurodegenerative conditions due to their strong association with increases in oxidative stress. Many of these same enzymes are also associated with tumorigenesis, invasion, or migration.

The selected enzymes for this paper appear not only sensitive to oxidation, but also key players in the underlying pathologies and in some cases, genetic causes of disease. It would appear that while nature has taken advantage of the reactivity of the sulfur group within cysteine to help regulate the response to oxidative stress, it also leaves these enzymes vulnerable to chronic conditions that promote prolonged exposure to an oxidizing environment. Thus, as our antioxidant defenses decline over time and cellular exposure to oxidizing conditions is increased, either through metabolic activity of the mitochondria, or by exposure to oxidizing environmental agents, this subset of cysteine-dependent enzymes become increasingly inhibited. Such inhibition is expected to contribute to and promote neurodegeneration, with variable effects on cancer. This paper has only highlighted some of the significant cysteine-dependent enzymes that have been shown to be related to neurodegenerative diseases and not all of the tremendous efforts of the many researchers that have contributed have been referenced here.

## Figures and Tables

**Table 1:** Examples of cysteine-dependent enzymes that use cysteine within their catalytic site within the various domains as delineated by the enzyme commission categories.

Class 1	Class 2	Class 3	Class 4	Class 5	Class 6
Oxidoreductases	Transferases	Hydrolases	Lyases	Isomerases	Ligases
Protein-disulfide reductase	Mercaptopyruvate sulfurtransferase	PTEN	MerB^∗^	Protein disulfide isomerase	Parkin
Peroxiredoxin	Akt	Ubiquitinyl hydrolase 1	LuxS^∗^	GluRS^∗^	
Glyceraldehyde-3-phosphate dehydrogenase	Janus kinase 2	Histone deacetylase			
SOD1	Sulfurtransferase	PTP1B			
ALDH1L1	Epidermal growth factor receptor				
Tyrosine hydroxylase					

*For some enzymes above, data suggests that they are oxidized and that the cysteine is essential for activity but may or may not be considered part of the catalytic site in all species.

Note: Other enzymes are not listed here although they depend upon cysteine for activity; often such cysteines are linked to a structural requirement such as a disulfide bond rather than as part of a catalytic domain.
